# “It’s changed my life not to have the continual worry of being warm” – health and wellbeing impacts of a local fuel poverty programme: a mixed-methods evaluation

**DOI:** 10.1186/s12889-022-12994-4

**Published:** 2022-04-19

**Authors:** Alexandra Sawyer, Nigel Sherriff, David Bishop, Mary Darking, Jörg W. Huber

**Affiliations:** 1 School of Sport and Health Sciences, University of Brighton, Falmer, BN1 9PH England; 2 East Sussex County Council, Lewes, County Hall, St Anne’s Cres, Lewes, BN7 1UE UK; 3 School of Applied Social Sciences, University of Brighton, Falmer, BN1 9PH England

**Keywords:** Fuel poverty, Energy efficiency, Health, Wellbeing, Psychosocial, Mixed-methods, Deprivation, Inequality

## Abstract

**Background:**

Living in a cold home and being fuel poor can contribute to adverse physical and mental health. Energy efficiency interventions are considered the simplest ways of tackling fuel poverty and preventing associated negative health, wellbeing, and socio-economic consequences. The overall aim of the current study was to provide a greater understanding of the impact of a locally administered programme, which funded the installation of major heating/insulation measures in areas of high fuel poverty, on the health and wellbeing of beneficiaries of the programme.

**Methods:**

A mixed-methods approach to explore the health and wellbeing impacts of a fuel poverty programme in East Sussex that took place between October 2016 and March 2018. Beneficiaries completed the Warwick-Edinburgh Mental Wellbeing Scale before and after any heating/insulation work had been completed in their home. Beneficiaries were also asked to retrospectively rate their health pre- and post-installation. Interviews with 23 beneficiaries of the programme were conducted to explore in-depth the impact of the programme on people’s health and wellbeing and the wider social determinants of health.

**Results:**

A major heating/insulation measure was installed in 149 homes. The majority of measures installed were boilers (57.7%) and new central heating systems (32.2%). Self-rated health and wellbeing were significantly higher post-installation. Interviewees described clear examples of the positive impacts on physical health and wellbeing such as fewer chest infections, reduced pain, feeling less anxious and depressed, and generally feeling happier and more relaxed. Interviews also highlighted broader areas of impact such as reduced social isolation and increased use of domestic space. Many of the beneficiaries also reported a reduction in their energy bills since their new heating systems had been installed.

**Conclusions:**

The findings from the evaluation suggest that the installation of major heating or insulation measures such as new boilers have substantial benefits for the health and wellbeing of beneficiaries. The findings also suggest that the programme had a positive impact on wider determinants of health including reduction in stress and isolation that are likely to be part of the pathways between fuel poverty interventions and mental and physical health outcomes.

## Introduction

Fuel poverty is defined broadly as the inability to afford an acceptable level of warmth in the home and is determined by three principal factors including the energy efficiency of the property, energy costs, and household income. It is a relatively new concept which has received considerable attention in the UK, but is now also recognised across the world as a particular issue of poverty or deprivation, and often referred to as energy poverty or energy deprivation [1, 2]. In England, fuel poverty is now measured using the Low Income Low Energy Efficiency (LILEE) indicator [3] rather than the Low Income High Cost Indicator. The LILEE indicator classifies a household as fuel poor if they are living in a property with a fuel poverty energy efficiency rating of band D or below, and when they spend the required amount to heat their home, they are left with a residual income below the official poverty line. The most vulnerable groups to fuel poverty include older people (65 and older), single parents with dependent children, families who are unemployed or on low income, children and young people, pregnant women, people with disabilities, people with existing illnesses and long-term conditions, and single unemployed people [4]. Fuel poor households are more likely to live in energy inefficient homes across all tenures compared to non-fuel poor households. However, private tenants are at the greatest risk of severe fuel poverty due to lower incomes compared to owner occupiers and living in less energy efficient homes compared to social housing tenants [5].

Fuel poverty and living in a cold home is associated with poor physical and mental health. The Marmot Review into the health impacts of cold homes and fuel poverty found a strong association between cold temperatures and cardiovascular and respiratory diseases. Links between cold housing and minor illnesses such as colds and flu were also reported [6]. Fuel poverty and living in a cold home has also been linked to excess winter deaths, the phenomenon where frequency of death is higher in winter months than at other times of the year. In the UK, it has been estimated that a fifth of excess winter deaths are attributable to the coldest quarter of homes and that approximately 10% of excess winter deaths are directly attributable to fuel poverty [6, 7]. Living in a cold home can be stressful for many reasons (such as continued thermal discomfort and financial worries) and there is evidence internationally highlighting the negative impact of fuel poverty on mental health and wellbeing [8–10]. There can also be a significant amount of stigma attached to those living in fuel poverty [11]. This can be expressed in people feeling too embarrassed by their home to accept visitors, which can leave people vulnerable to loneliness and social isolation [12]. A recent study reported that embarrassment may also prevent people seeking assistance to improve their situation either from friends and relatives, or through support agencies [11]. Other wider social impacts of living in a cold home include tension amongst household members and restricted use of living space [13].

It has been suggested that energy efficiency measures and interventions are the simplest ways of tackling fuel poverty and preventing its related negative health, wellbeing and socio-economic consequences [14, 15]. In 2015, the government introduced a fuel poverty target for England to improve as many fuel poor homes as is reasonably practicable to a minimum energy efficiency rating of Band C, by the end of 2030 [16]. A national consultation, which covered issues such as measurement, targets, and vulnerability, was undertaken in 2019 and informed England’s current fuel poverty strategy [17]. Energy efficiency measures such as insulation, double glazing, and heating improvements aim to reduce energy demand making it more affordable to keep homes warm. Evidence suggests that energy efficiency interventions targeted at those at risk of fuel poverty and living in poor quality housing may lead to health improvements. In particular, reviews have shown that energy efficiency interventions can improve general, respiratory, and mental health outcomes [18, 19]. Reviews also suggest a range of socioeconomic outcomes linked to warmth and energy efficiency improvements such as reduced fuel bills and less time off from work/school [18, 20]. Data from qualitative research reviewed suggest that improved thermal comfort resulted in more usable space indoors, improvements in diet, improved household and family relationships, and more opportunities for leisure and studying. There is also evidence that energy efficiency interventions are more effective if at-risk groups are targeted. For example, a meta-analysis found that significant health benefits from energy efficiency interventions were identified for vulnerable groups as a whole (e.g. children, the elderly, those on low incomes or with pre-existing medical conditions) and for children and people in poor health in particular. Recipients on low incomes saw the greatest improvements in health following energy efficiency interventions [19].

In East Sussex 8.2% of households are fuel poor compared with 7.5% in the South East and 13.4% in England. However, there is variation within East Sussex [21]. For example, the proportion of fuel poor households in Hastings is 10.8%, compared to 8.5% in Eastbourne. This has been attributed to the nature of the housing stock but also as a result of poor housing management in some areas and many households being on low incomes in Hastings. In October 2016, NHS Hastings and Rother Clinical Commissioning Group (H&R CCG) established an 18-month pilot project, known as the Healthy Homes programme, to fund installation of major heating and insulation measures, through the Winter Home Check Service (WHCS). The WHCS was commissioned by East Sussex County Council’s (ESCC) Public Health team and offered advice, home visit assessments, provision of small preventative measures and the coordination of installation of major heating/insulation measures (where funding allowed). The programme was targeted at poor condition properties in the private sector (owner-occupiers and private tenants) where fuel poverty was an issue due to unsatisfactory heating, poor thermal insulation, and generally poor energy efficiency. Major measures funded by the programme included: cavity wall insulation, hard-to-treat cavity works, loft insulation, floor insulation, solid wall insulation, full central heating systems, central heating boiler replacement, and storage heaters. The programme reached 149 properties between October 2016 and March 2018 in wards with the highest number of fuel poor households in Hastings and Rother.

The overall aim of the current study was to provide a greater understanding of the impact of this locally administered programme, which funded the installation of major heating/insulation measures in areas of high fuel poverty, on the health and wellbeing of beneficiaries of the programme.

## Methods

### Design

The study was based on a mixed-methods approach, drawing on organisational monitoring and primary research data, with before and after data collection points, as part of an evaluation of the NHS Hastings and Rother Clinical Commissioning Group (H&R CCG) fuel poverty programme. The use of organisational monitoring data was motivated by a desire to reduce data burden for vulnerable participants. The evaluation utilised both process and impact/outcome measures; this paper reports only on the health and wellbeing outcomes.

### Data collection

Data collection was carried out via three main phases: 1) Baseline survey data collection; 2) Follow-up survey data collection; and 3) Follow-up semi-structured interviews with beneficiaries of the programme and key stakeholders (this paper only presents findings from the interviews with the beneficiaries). Baseline (pre-intervention) data were collected before any heating and/or insulation work had started. Follow-up (post-intervention) data were collected after all heating and/or insulation work was completed.

### Survey data

Survey data (collected between October 2016 and July 2018) was derived from organisational monitoring data and questionnaires administered by the providers of the WHCS. This data was incorporated into the study design as secondary data that were analysed by the research team.

Baseline survey data collection *-* the following information was collected at baseline by an energy assessor (a qualified energy advisor who investigates the physical aspects of the property and the heating and water systems of the property, also providing advice on behaviours that will both promote health and wellbeing, including energy efficiency advice) at the first home assessment visit of all beneficiaries:

- Data about scheme beneficiaries – local authority area, sociodemographic information, household income, current health, disability, details of benefits, carer status;
- Data about scheme beneficiaries’ homes - household size, detachment type, property type, tenure type, number of bedrooms, number of occupants, storeys, main fuel type, number of rooms with no heating, type of heating, whether boiler was working at the time of assessment, age of boiler, property Standard Assessment Procedure (SAP) rating;
- Referrals – source of referrals e.g. landlord, support service, GP, family/friend, self-referral;
- Wellbeing – All beneficiaries were asked to complete the Warwick-Edinburgh Mental Wellbeing Scale [22] to assess mental wellbeing. The WEMWBS is a 14-item questionnaire, with five response categories ranging from (‘none of the time’ (1) to ‘all of the time’ (5) and is scored by summing all the items into a total wellbeing score (range 14–70). A sample item is ‘I’ve been feeling optimistic about the future’. The WEMWBS has been shown to have good validity, internal consistency and test–retest reliability with a large general population sample [21].

### Follow-up survey data collection

The following information was collected after the heating/insulation works had been installed:

- Data on scheme interventions - type of advice given, whether a minor measure (e.g. draught proofing, gutter clearance) was also installed, type of major installation, cost of installation, property SAP rating. This information was completed by the energy assessor and/or the WHCS service provider;
- Beneficiaries’ subjective experiences with service - a range of questions were included in a “Post Installation Customer Handover Checklist” to measure satisfaction with the service (e.g. How would you rate the overall quality of the service?). This questionnaire was posted to beneficiaries approximately 6 weeks after the measure was installed;
- Health and wellbeing - Two single item questions were asked to measure beneficiaries’ health in the post-intervention phase only (In general, how would you describe your health prior to the preventative works being complete? - excellent, very good, good, fair, poor and in general, how would you describe your health now? excellent, very good, good, fair, poor). These two questions were included in the “Post Installation Customer Handover Checklist”. The WEMWBS was also completed by beneficiaries post-intervention. These health and wellbeing questionnaires were planned to be completed approximately 6 weeks after the measure was installed; initially some beneficiaries were contacted too early.

### Interviews with beneficiaries of the healthy housing programme

Interviews were carried out with beneficiaries of the programme between February and June 2018. All beneficiaries who had a major heating or insulation measure funded by the NHS H&R CCG Healthy Homes programme were eligible for participation in the qualitative interviews. Beneficiaries did not need to have completed pre- and post-intervention surveys in order to be eligible for the interviews. In addition, participants had to be over 18 years of age; be able to give informed consent; and be able to understand and speak English coherently. Study packs were posted to beneficiaries of the Healthy Homes programme by the provider of the WHCS. Packs comprised: a letter introducing the study, a participant information sheet, and a reply slip to indicate interest in participating. Following receipt of a completed reply slip, a member of the research team contacted any beneficiaries who had responded positively, clarifying that they understood the nature of their involvement, and if they agreed, arranged a suitable date and time for interview. A reminder letter was sent to any beneficiaries who had not responded approximately 2 weeks after the first letters of invitation had been sent.

A semi-structured interview schedule was used to generate qualitative data, which allowed participants to have flexibility in their answers and identify or explore further areas as required. Topics included: experience of the application process, experience of the assessment process, experience of the installation, impact of the heating/insualtion intervention on health and wellbeing, and overall satisfaction. In addition, a simple and short structured questionnaire was administered to gather basic socio-demographic characteristics (e.g. age, ethnicity, education) and property/household characteristics (e.g. household size, number of rooms, property type, tenure type, main type of fuel). One hundred forty-eight beneficiaries (one of the beneficiaries had died since the installation hence the lower number of invitees) were invited to take part in the interview and 26 people returned a reply slip to indicate they would be interested in participating and 23 were subsequently interviewed (16% response rate). This is a reasonable response rate considering the vulnerable population and is similar to other studies which have used an opt-in recruitment procedure. Interviews lasted approximately 30 min and interviews either took place at the participant’s home (*n* = 12) or over the telephone (*n* = 11). Participants were given a £10 ‘thank you’ voucher for their time.

### Ethical approval

The University of Brighton’s Life, Health and Physical Sciences Cross-School Research Ethics Committee (CREC) reviewed and approved this evaluation.

### Data analysis

#### Survey data

To safeguard data quality, the anonymised survey data was checked for the following: double-checking coding of observations or responses and out-of-range values; checking data completeness; adding variable and value labels where appropriate; double entry of data; statistical analyses such as frequencies, means, ranges or clustering to detect errors and anomalous values. Basic descriptive quantitative analysis was then conducted on the secondary data provided. Graphs were used to illustrate the main findings. Data was also analysed to explore the impact of the scheme and to understand the impact for different groups of target beneficiaries. SPSS data analysis software (Version 24) was used for all analysis. Normality tests were performed on the data prior to running the analysis. Difference in pre and post WEMWBS scores were normally distributed, therefore parametric tests (paired samples t-tests) were used. Difference in pre and post self-rated health were not normally distributed, therefore the parametric t-test was conducted with bootstrapping. To explore the impact of the scheme on health and wellbeing for different groups of target beneficiaries repeated measures ANOVA was used. The interaction term of the repeated measures ANOVA was explored for each analysis to understand how different characteristics of the scheme/beneficiaries impacted on wellbeing (a significance value of *p* < .05 indicated a significant interaction).

#### Interview data

The evaluation team as a whole were responsible for the analysis of the interview data. With participant’s permission, all interviews were audio recorded, quality checked, and fully transcribed. Qualitative thematic analysis was used to inductively (from the data) and deductively (based on the project’s objectives and indicators) analyse the data. Braun and Clarke’s [23] method was used to identify, describe, and analyse themes and patterns within the data. After transcripts were read and re-read to become familiar with the data, interviews were coded to generate an initial pool of codes. Codes were then collated into potential themes. Themes were reviewed by three authors (AS, JH, NS) in relation to the generated pool of codes and the entire data set. Finally, definitions and names were generated for each theme. Specialised qualitative data software (NVivo; Version 11) was used to support this process. Adopting a team approach, analytical processes were triangulated to increase reliability and validity of the findings. Direct quotes are referred to by participant codes to ensure anonymity.

## Results

### Scheme beneficiary and property characteristics

A major heating and/or insulation measure was installed in 149 homes as part of the Healthy Homes programme. Table 1 displays the main demographic and property characteristics of programme beneficiaries. Ages of beneficiaries ranged from 22 to 94 with the average age being 57.7 (SD = 17.5). All but three beneficiaries had a household income of under £16,000. Of the beneficiaries of the Healthy Homes programme approximately 90% described living with a long-term health condition and 21% were living with a disability, 20% of families had a child aged 16 years or under, and 20% were 75 years or older.

**Table 1:** Demographic and property characteristics of scheme beneficiaries

	Scheme beneficiaries ***N =*** 149 (%)	Beneficiaries who completed pre and post WEMWBS ***N*** = 78 (%)	Beneficiaries who participated in the interview ***N*** = 23 (%)
**Gender**^**a**^			
Male	47 (32)	22 (28.6)	5 (21.7)
Female	100 (68)	55 (71.4)	18 (78.3)
**Ethnicity**^**a**^			
White British	125 (96.9)	64 (97)	23 (100)
Other	4 (3.2)	2 (3)	
**Employment status**			
Employed full time	5 (3.4)	2 (2.6)	1 (4.3)
Employed part time	6 (4.0)	2 (2.6)	0 (0)
Unemployed	87 (58.4)	45 (57.7)	10 (43.5)
Self-employed	2 (1.3)	2 (2.6)	1 (4.3)
Retired	49 (32.9)	27 (34.6)	11 (47.8)
**Property type**			
Bungalow	8 (5.4)	7 (9)	(0)
Flat/Maisonette	99 (66.4)	50 (64.1)	16 (69.6)
House	42 (28.2)	21 (26.9)	7 (30.4)
**Occupants**^**a**^			
1	82 (55.4)	45 (57.7)	18 (78.3)
2	38 (25.7)	19 (24.4)	4 (17.4)
3	16 (10.8)	6 (7.7)	0
4	8 (5.4)	8 (10.3)	0
5+	4 (2.7)	–	1 (4.3)
**Tenure**			
Owner occupier	58 (38.9)	32 (41)	15 (65.1)
Privately rented	91 (61.1)	46 (59)	8 (34.8)
**Detachment type**			
Terraced	92 (61.7)	45 (57.7)	–
End of terrace	15 (10.1)	10 (12.8)	–
Semi-detached	26 (17.4)	14 (17.9)	–
Detached	14 (9.4)	8 (10.3)	–
Other	2 (1.3)	1 (1.3)	–
**Number of storeys**			
1	99 (66.4)	54 (69.2)	–
2	35 (23.5)	19 (24.4)	–
3	13 (8.7)	4 (5.1)	–
4	2 (1.3)	1 (1.3)	–
**Main fuel type**			
Electric	43 (28.9)	25 (32.1)	2 (8.7)
Gas	104 (69.8)	53 (67.9)	21 (91.3)
Oil	2 (1.3)	–	0
**No working boiler**	103 (69.1)	53 (67.9)	–
**Rooms with no heating**	68 (45.6)	34 (43.6)	–

Note. ^a^missing data (*N* ranges for full sample from 129 to 148), − not measured in participants who took part in interviews. On occasions the percentages may not add up to 100% precisely due to the rounding up or down of decimal places

The majority of measures installed were new boilers (57.7%) and central heating systems (32.2%). Other works included storage heaters (6.7%) and loft insulation (3.4%). In addition to the installation of major measures, 58 properties (38.9%) also had some minor heating/insulation measures installed. These included but are not limited to: boiler service/repair, gutter clearance, draught proofing, and door/window repairs.

### Impact on health and wellbeing

The WEMWBS was used to assess wellbeing before and after the installation of major measures. Of the 149 homes which received a major heating and/or insulation measure, 78 beneficiaries (representing 52.3% of all scheme beneficiaries) completed the WEMWBS before and after the installation. This response is similar to the response rate reported by an evaluation of a recent large-scale energy efficiency intervention [24]. Table 1 displays the characteristics of the beneficiaries who completed the WEMWBS at both time points compared to the overall scheme beneficiaries and these seem to be broadly similar. Ages of participants who completed the WEMWBS ranged from 22 to 93, with the average age being 60.5 (SD = 16.8).

**Fig. 1:**
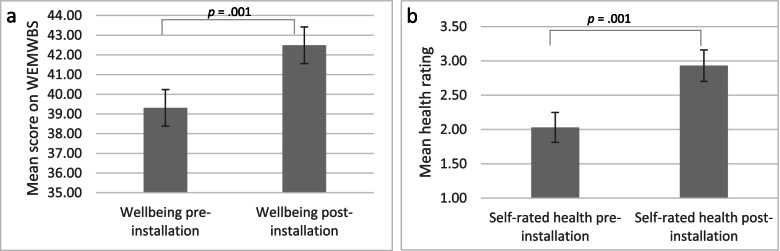
**a**. Scores on WEMWBS pre- and post-installation (higher scores indicate better wellbeing). **b**. Retrospective self-rating of health pre- and post-installation (ratings 1–5 with 1 indicating poor health and 5 indicating excellent health)

Figure 1a displays the mean scores of the WEMWBS pre- and post-installation. On average people experienced higher wellbeing post-installation (M = 42.49, SD = 9.83) compared to pre-installation (M = 39.31, SD = 11.06). This difference was significant, t(77) = 3.42, *p* = .001, and represents a medium-sized effect (r = .36, d = .39). However, scores on the pre- and post-WEMWBS are considerably lower than the wider UK population norm of 49.9 [25] and that reported in Hastings, 48.50.^1^ Figure 1b displays the responses to the two single questions (“In general, how would you describe your health prior to the preventative works being completed?” and “In general, how would you describe your health now?”) about beneficiaries’ health prior to the installation (retrospective assessment) and post-installation. One hundred one people completed this question at both time points. On average people reported better health post-installation (M = 2.93, SD = 1.16) compared to pre-installation (M = 2.03, SD = 1.11). This difference was significant, t(100) = 9.29, *p* = .001, and represents a large-sized effect (r = .68, d = .92).

**Fig. 2:**
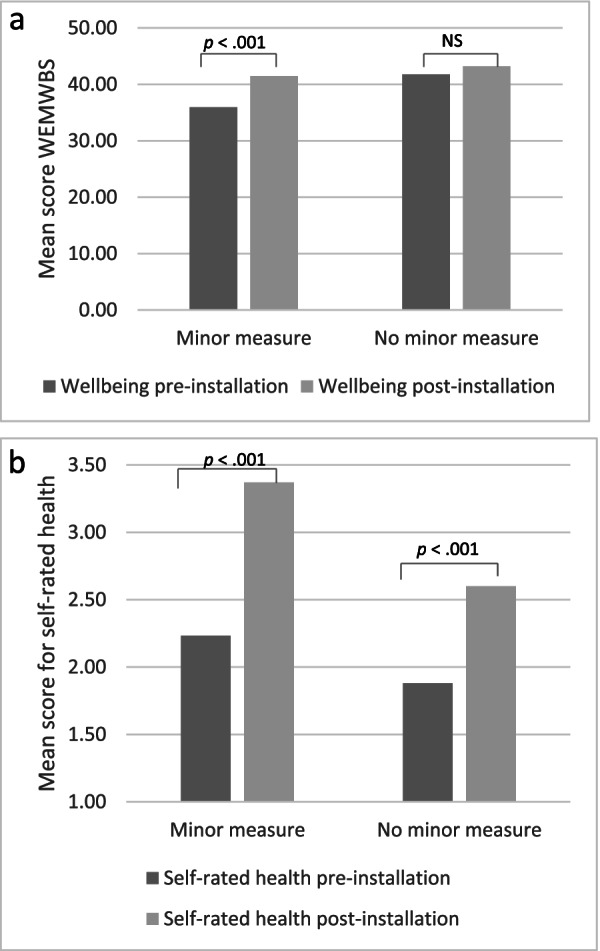
**a**. Impact of minor measures on wellbeing. **b**. Impact of minor measures on self-rated health

Data was also analysed to explore the impact of the scheme on health and wellbeing for different groups of beneficiaries, property characteristics, and intervention characteristics. Overall, beneficiary characteristics, property characteristics, and intervention characteristics did not impact on pre- and post-wellbeing or pre- and post-self-rated health. However, there was a significant interaction between minor measures and pre- and post-wellbeing (*p* < .05) and between minor measures and pre- and post-self-rated health. Figure 2a and b display this relationship. In particular, those who had a minor measure installed in addition to a major measure reported greater increases in wellbeing (*F*(1,76) = 4.99, *p* < .05) and self-rated health (*F*(1,99) = 4.65, *p* < .05) from pre- to post-intervention. This finding suggests that those who had a minor measure installed reported a larger increase in wellbeing and health scores compared to those who did not, suggesting that a combination of both a minor and major measure has a greater impact on wellbeing and health. However, as this is not a controlled study, other variables could influence this finding (e.g. tenure, property type).

### Interviews with beneficiaries

Interviews were conducted with 23 beneficiaries of the scheme to generate in-depth primary data regarding the impact of the scheme and develop a more nuanced understanding of beneficiaries’ perceptions of project impacts. Table 1 displays the demographic characteristics of the sample of beneficiaries who were interviewed. Ages of participants ranged from 33 to 87 (*M* = 61.5, *SD* = 15.9). The characteristics of the sample were broadly similar to the beneficiaries of the Healthy Homes programme overall, although a larger proportion were owner-occupiers. Participants self-reported numerous health conditions such as diabetes, cardiovascular disease, respiratory problems, mental ill health, cancer, Raynaud’s condition, and arthritis, all of which could be worsened by living in a cold home. Postcode data were analysed using the indices of multiple deprivation (IMD) from the Office of National Statistics to gain an indication of the socioeconomic background of the interviewees. IMD scores range from 1 to 32,844 with a low score indicating most deprivation and a higher scoring indicating least deprivation. For the purpose of this research, IMD scores were categorised into quintiles to give an overview of the kinds of areas participants were drawn from. Of the people interviewed 70% lived in one of the 20% most deprived areas of England (see Table 2). Fourteen (61%) of the interviewees had a new boiler installed, seven (30%) had a whole new central heating system installed, and two (9%) had storage heaters installed as part of the programme. Ten interviewees reported that they also had some minor heating work completed as part of the WHCS such as draught proofing, new thermostats on radiators, energy saving light bulbs fitted, and aluminium foil fitted behind radiators.

**Table 2:** Index of multiple deprivation (IMD) based on postcode data

IMD Quintile	***N*** (%)
Band 1 (1–6568) – most deprived	16 (70)
Band 2 (6569–13,137)	4 (17)
Band 3 (13138–19,706)	3 (13)
Band 4 (19707–26,275)	0
Band 5 (26276–32,844) – least deprived	0

The section below presents the findings of the interviews conducted with 23 beneficiaries of the H&R CCG Healthy Homes programme, focussing on the perceived impacts of the programme. All beneficiaries interviewed reported positive impacts of having new heating measures installed in their homes. These impacts were broad and included: i) Thermal impacts; ii) Physical health impacts; iii) Psychological wellbeing impacts; iv) Psychosocial impacts; and v) Financial impacts. Before these are described in detail, people’s motivations for applying to the WHCS are summarised to help understand the context of these reported impacts. The primary reason people applied to the scheme was that they were either currently cold in their home, or they were worried about being cold in the future. It was very common for people to report being worried and concerned about how they were going to cope with upcoming winters with their old heating systems. Two beneficiaries reported not having any heating in their home at all, with one person stating that they “put the oven on for an hour to take the chill out” (B19, Male, central heating replacement, 60 years old). Many of the people who did have some form of heating described that it was not effective in keeping them warm. One woman, who was in her late 60s, only had a single oil-filled radiator in her two-bedroom flat and this was in the living room, which meant this was the only room that had any heating:

“All I had was an oil-filled radiator, and I have got Parkinson’s…And I suppose it was that I was feeling the cold more, and I thought I have got to do something about this”. (B4, Female, central heating, 68 years old).

Many of the people who did have some form of heating described their heating systems as being old and faulty, with one person describing that their boiler broke down as frequently as a couple of times a week. This meant that some of the people interviewed reported regularly having no heating or hot water. Many people reported using alternative strategies to stay warm. These included using hot water bottles, wearing hats and gloves indoors, using portable heaters, and only heating one room. For example, one couple who lived in a privately rented flat had a very old and temperamental boiler, which meant they struggled to keep warm. They reported that when they were inside they had to dress like they were going “out in the snow”. Two of the people interviewed lived on the seafront and they reported that the cold was exacerbated by the coastal weather conditions, making it particularly uncomfortable:

“I live in a flat on the seafront, and believe me when the wind comes off, straight onto these houses, it’s like being able to have a free wash and blow dry because the rain would come in and drip down. So that was the wash, and then I’d step back to the wind, to blow dry my hair”. (B20, Female, storage heaters, 61 years old)

Some people also reported that not only were their heating systems not working properly but they also had concerns over the safety of their boilers. For example, one man described that when he turned on his 62-year old boiler he experienced gas blow backs from the boiler, which made him feel dizzy. Another woman, who also has four young children living at home, described how her old boiler was leaking carbon monoxide:

“They said our boiler was probably one of the worse they’d seen, I think it was 30 years old and it was leaking carbon monoxide. So it really needed to be changed…either the thermostat didn’t work, it didn’t heat, we were using electric heaters, like the really cheap fan heaters, which again cost a fortune to run”. (B5, Female, new boiler, 42 years old)

The majority of participants said that they would not be able to afford to pay for a new central heating system or replacement boiler themselves, and for those people who lived in private rented accommodation it was often difficult to get landlords to fix the heating:

“There was no window in my downstairs toilet for five months, they didn’t even come and board it up, I had rain pouring through the ceiling. It was bad, the house was really bad, I think there was about thirty repairs that needed doing”. (B16, Female, new boiler, 52 years old)

As well as concern over inadequate heating people also reported applying to the scheme because they noticed that their fuel bills were very expensive and they wanted advice on how these could be reduced. Many of the people reported how expensive it was to heat their home. For example, some of the people interviewed were aware that that their heating systems were inefficient, which is why their fuel bills were higher than expected. One person described how his gas bills were extremely high even in the summer when he did not use any heating.

### Thermal impacts – “it’s the first time it’s ever been that warm”

As described earlier many of the people interviewed were either living with no heating or inadequate heating. Therefore, as might be expected, the majority of participants reported feeling warmer since their new heating systems had been installed in their home. There was a significant cold spell in the winter of early 2018 and many of the beneficiaries commented that they were very happy to be able to have adequate heating and warmth during this period.

“To be warmer is lovely I have to say, because I can’t deal with the cold at all. And neither can my husband as he’s got older. To have that little extra bite of heat is gorgeous”. (B2, Female, new boiler, 60 years old)

“That cold spell we had with the snow when it got bitterly cold, to have that warmth when I needed it, to be able to put it on and have the whole flat beautifully warm. I think it’s the first time it’s ever been that warm, it’s been marvellous”. (B11, Female, central heating, 87 years old)

Those people who also had children living at home reported that their children also feel warmer now, which for them was the most important impact:

“The children are warm, which I suppose is probably the biggest thing”. (B5, Female, New boiler, 42 years old)

It was also common for people to report that they generally felt more comfortable since having their new heating installed. Many people reported that because they were warmer they no longer needed to use strategies to keep warm, such as wearing extra blankets or layers of clothes when they were in the house:

“It [new boiler] has just made life more comfortable for me, all round. Last year I was putting on all sorts to keep myself warm”. (B3, Female, new boiler, 82 years old)

“I don’t have to overdress in the house. Before I’d have a jumper on, t-shirt on, pyjamas on top of that, or underneath it, dressing gown, you know, a blanket to sit with. I don’t need to do as much as that”. (B16, Female, new boiler, 52 years old)

Several people also reported that the damp in their home had also improved because it was warmer:

“There’s definitely an improvement. I think because the flat was a little bit damp before, so now that I’ve got proper heating in every room, it’s getting rid of the damp and that’s really helping”. (B18, Female, central heating, 45 years old)

Finally, not only did people report that they are warmer now but that they could also get warm water whenever they want, something which is required throughout the year, not only during cold months.

### Physical health impacts – “I am not in so much pain”

Many of the people interviewed described numerous positive impacts on their physical health, which they directly attributed to having improved heating in their homes. Firstly, there were frequent examples where people reported fewer health problems such as chest infections, pneumonia, and colds, compared to when they had their old heating system:

“The thing is before when I got pneumonia it was around Christmas time in January, but this winter I would say I have not, unlike all my friends who have had colds or had flu or whatever, I have not had the slightest bit of a cold or slightest bit of pneumonia or anything at all, I’ve kept well all the time touch wood. Touch wood I’ve had a completely illness free winter”. (B9, Female, New boiler, 76 years old)

“It's made it better in that, like I got chest infections regularly anyway, but my chest infection wasn’t as bad this year as it was last year, so it wasn’t as bad after the [new] boiler”. (B8, Female, new boiler, 33 years old)

Several of the beneficiaries also reported less pain now that they were warmer. This was particularly evident for those people who suffered from arthritis. The two quotes below illustrate how the cold worsened one person’s arthritis and also how having a warm home can alleviate arthritic pain:

“I suffer from arthritis and when it gets cold that’s when the pain comes in my hands. Bad. And my legs. And I used to phone my daughter. She goes, “What’s the matter?” Cos I was crying. I goes, “I can’t cope with this. It’s so bloody cold. It’s making all my legs really, really hurt.” (B22, Female, Storage heaters, 69 years old)

“I have got very bad arthritis everywhere, and if I am warm I am not in so much pain. So it’s [warmth] helped everyway.” (B3, Female, new boiler, 82 years old)

Another person, who suffers from a range of health problems, describes how being warmer has reduced the pain and suffering she experiences when cold. She goes on to say later in the interview that she has taken fewer painkillers than she did last year:

“One of my biggest problems is temperature control, my internal thermostat just doesn’t work, so if I get cold, it’s really hard for me to warm up and if I get cold it increases my pain levels. So the fact that I’ve been able to stay warm this last winter, has probably, overall, reduced my level of suffering, because I haven’t been freezing cold and therefore in more pain”. (B17, Female, central heating, 39 years old)

Another woman described that she is also visiting her GP less frequently because she is in less pain:

“I broke my leg last year, in two places, so I’ve got metal pins in my leg, and when it was cold, the year before, I always thought it was an old wives tale, that when it’s cold, it affects your bones, but of course having metal pins, it really did affect the pain, so I was sort of going up and down to my doctor”. (B13, Female, new boiler, 59 years old)

Several of the people interviewed also described that being warmer in their home has meant they can now move around more, which has the positive effect of reducing their pain:

“Yeah because my muscles hurt sometimes, really, really bad they hurt and if the heating’s on, and they’re playing up, I can walk a bit”. (B16, Female, new boiler, 52 years old)

Two people also reported that their children’s health is better. Specifically, the woman below describes that she can now bathe her daughter regularly, due to having hot water and being warmer when she comes out of the bath, which has had a positive impact on her young daughter’s eczema:

“The other aspect is that my daughter, she’s got eczema, and so I like her to have a bath at least every other day so that I can apply her cream that she gets from the doctor. It was just such a pain with the old boiler because it cooled down so quickly but I couldn’t then top it up with hot water, because it would come out cold for the first five minutes. So now she’s having more regular baths and her eczema’s a lot better, because I’m able to keep on top of her treatment”. (B17, Female, central heating, 39 years old)

Finally, this same participant discussed that having no access to hot water was impacting her personal hygiene. Although this was only mentioned by one person this impact of having no hot water and heating is significant because of the associated negative physical (and social impacts):

“It’s helped me with hygiene and things, because obviously, I wasn’t comfortable having a shower before, because it switched off and I’m suddenly freezing cold with shampoo in my hair or whatever, well I just didn’t do that anymore. I was having a bath maybe once a week or ten days or so and probably not washing my hair and things…I’m actually a lot more hygienic”. (B17, Female, central heating, 39 years old)

### Psychological wellbeing impacts – “It’s just made my life so much easier”

Many of the interviewees also reported that having the new heating measures had a positive impact on their mental wellbeing which was attributed to a range of different factors. For example, many participants reported anxiety about their old boiler breaking permanently, not being able to afford to fix it and being left without heating and getting cold. Having a new and working heating system helped alleviate a lot of this worry and anxiety:

“Last winter I thought, I can’t go through another winter like this. But I just knew I couldn’t afford it to have it done”. (B4, Female, central heating, 68 years old)

“It’s stress and strain off me because I am confident that it’s new and it’s going to work”. (B3, Female, new boiler, 82 years old)

Another woman who was living in a rented property described the worry of having to look for somewhere else to live with her young daughter because of having no heating or hot water, something that she no longer has to think about:

“Although I think my landlord would have dealt with it, I don’t think he would have immediately dealt with it and I would have had to have found somewhere to go, because I couldn’t have stayed there, if there was no heating or hot water. I don’t worry about that now, so that’s less stressful. It was always the question, can I even stay here? Do I need to actually find somewhere else to live, because this is really becoming a problem…I don’t have to worry about that now. So it’s had a direct impact on my mental stress levels as well, it’s really reduced that level of that particular kind of anxiety”. (B17, Female, central heating, 39 years old)

People’s sense of wellbeing also increased in numerous ways with one person describing “it’s made life a lot happier” (B21, Male, central heating, 54 years old). Some people described that living in a cold home can make them feel “ashamed”, and one man described having no heating as “psychologically degrading” (B6, Male, new boiler, 74 years old). The quote below illustrates how having a warm home has improved one woman’s sense of pride and self-worth:

“My dignity as well and my pride and my self-worth, because you feel almost like a homeless person if you’re in a cold house and you can’t feed yourself properly. There’s a lot of shame involved in that.” (B10, Female, New boiler, 37 years old)

Several people who lived with depression said that having a working heating system and being warm helped them with their mental health:

“Just wonderful knowing that they [engineers] were coming and then when it was installed, just the peace of mind, feeling faith with your boiler. It’s anyone’s biggest fear that the boiler’s going to go and it was just a wonderful feeling. I suffer from depression and that really lifted my spirits. I know it sounds daft, it used to be diamonds and pearls but now it’s my boiler”. (B13, Female, new boiler, 59 years old)

“The depression is much improved, just knowing that you haven’t got to go through the winter, thinking am I going to be able to afford this, will I have the money to do this? And being much more comfortable, without putting loads of blankets on you and feeling like a normal person does”. (B20, Female, storage heaters, 61 years old)

Many of the people living in fuel poverty who were interviewed had complex and chaotic lives where having no heating was described as one multiple challenges they faced such as physical health conditions, poor mental health, and not being able to afford food. Making people warmer is one way to relieve the stress and this is reflected in the quote below:

“it’s just made my life so much easier, because when you’re very, very vulnerable, depressed, and dealing with whatever health conditions you’re dealing with and you’re cold and you’re hungry, that pushes you right to edge. To be able to be warm, even if sometimes I was hungry this winter, it just made such a difference”. (B10, Female, New boiler, 37 years old)

Therefore, not surprisingly, several people described that the mental health impacts of having a warm home were the most significant to them:

“The mental side of the health side, because mentally it destroys you, if you’ve not got the proper things you need. I know it’s warm enough to turn the tap on and it’s hot. It’s like a big relief, instead of sitting there saying “oh I’ve got to go downstairs, boil the kettle ten times to put in the sink”, up and down the stairs, and don’t want to get out of bed because you’re shivering, because it’s cold, so I think the mental side of it.” (B16, Female, new boiler, 52 years old)

Finally, one impact of having a new heating system, which influenced people’s wellbeing was an increased sense of control. In particular, some people discussed feeling like they now had some control over their environment and that they could adjust their heating depending on how they felt due to the installation of a thermostat. This was especially important for people who previously had storage heaters, which did not allow you to adjust the temperature:

“Since I’ve had the new boiler, I am in control of the temperature…I’ve got it exactly how I need it to be”. (B17, Female, central heating, 39 years old)

### Psychosocial impacts – “My house became a home”

Some of the people interviewed also commented on the broader benefits of having heating works installed in their home. Firstly, some of the beneficiaries reported that the new installation opened up rooms that were previously unheated, therefore increasing the amount of space they could use in their home. For example, one woman described that before she had a new boiler installed she and her daughter would just stay in the living room but now the whole flat is warm they can move around more:

“I’m more mobile within the flat. So instead of us all huddling on the sofa with a duvet, I’m pottering around doing stuff”. (B17, Female, central heating, 39 years old)

Similarly, another woman described how she is now able to “live” in her flat and is no longer confined to a single room because her whole flat is warm. These impacts are best reflected in the quote below where a woman describes that her “house became a home”:

“I was cold, I had to have one room heated, and so I would have to put myself in one room, keep the door closed, and just heat one room. This winter I have been able to come in and out of rooms and have all the rooms warm... So I’ve been able to move around my flat, I’ve been able to live in it”. (B10, Female, new boiler, 37 years old)

Another significant impact described by one of the women interviewed is that she felt that she could now invite friends to her place. This is because not only is her flat warmer and so visitors would be more comfortable but also because previously she had felt ashamed about her situation and had felt too embarrassed to have people around. As such, she consequently reported feeling less lonely:

“I didn’t really have people over because of the shame of the situation, so it’s very valuable because I’ve had people over which has provided me with support and seeing people that I wouldn’t have had the winter before…people have been able to come over and sit and talk”. (B10, Female, new boiler, 37 years old)

Several beneficiaries described that having working heating gave them more freedom to decide whether to stay in or go out. For example, one person reported that because they knew their home was warm they were more likely to stay in rather than go out to find somewhere to get warm. On the other hand, several people described they feel they can now go out knowing they will come back to a warm home. Fundamentally what seems to be important is that people now feel they have some choice over what to do and no longer feel restricted by the temperature of their home.

“When I go out and I know I can come in at any time and get the flat warm, it’s much better”. (B18, Female, central heating, 45 years old)

### Financial impacts – “I’m actually in credit”

Many of the beneficiaries reported a reduction in their energy bills since having their new heating system installed. However, it should also be noted that for some participants it was too early to tell whether there would be an impact on their bills because they had yet to experience a full winter with their new heating.

“I reckon my gas consumption payment saving on just that period over the last twelve months, I would say I’ve saved about 25 per cent”. (B1, Male, new boiler, 63 years old)

“Big improvement. I’m actually in credit. That’s a first. I pay a sum monthly for gas and electric combined and I stuck to the same amount that I had been paying before which was actually 70 pounds a month”. (B11, Female, central heating, 87 years old)

This reduction in energy bills was primarily attributed to a more efficient heating system, which did not need to be on for as long in order to adequately heat up the home:

“We were spending a fortune to keep it so that it wasn’t freezing in here but because it warms it up properly now with like a little bit of use we have got a reduction in the heating bill because it doesn’t have to be on absolutely constantly”. (B8, Female, new boiler, 33 years old)

People reported that this reduction in their energy bills relieved financial pressure, which is especially significant for people who are already living on an extremely limited income:

“I’m mainly on Incapacity Benefits, so I don’t get a lot of money as you can imagine, and I have to sort of break it down into bills and things like that, so it has made it a heck of a lot easier”. (B21, Male, new boiler, 54 years old)

“My bills have been less. I can’t even afford to feed myself at the moment, I’m taking food vouchers, but as a result of the work that they’ve done for me, it’s ongoing supported me, because all my bills have been so much lower”. (B11, Female, central heating, 87 years old)

However, it is also important to highlight that two people reported an increase in their bills. One person noted that their bills were higher since the installation of the new boiler which was attributed to keeping the heating on for longer compared to their old boiler:

“This time of year we’re keeping the heating on longer. And before we had the new boiler I was inclined to turn the heating off for economy. And now I turn it down when it’s warmed up and I forget about it. So obviously it’s on longer”. (B2, Female, new boiler, 60 years old)

Finally, another beneficiary noted that some of their benefit entitlements had been reduced since the new boiler was installed, and consequently they cannot afford to heat their home as much as they would like, thus highlighting again the complex lives of many of the interviewees.

## Discussion

Findings from the service monitoring data suggest that beneficiaries experienced improved health and wellbeing following installation of major heating/insulation measures. These findings were corroborated in the interviews with beneficiaries where clear examples of the positive impacts on physical health and wellbeing were identified. This improvement in health and wellbeing is in line with previous reviews of energy efficiency interventions [18–20].

Despite these promising findings it is important to note that the evidence regarding the impact of energy efficiency interventions on health is conflicting with many studies reporting no impact [14]. These inconsistent findings can be explained partly by the considerable variation in the type, delivery, and length of interventions received by participants across the different studies, as well as the differing measures used to assess health impacts. For example, one meta-analysis found that studies which used medical tests to assess health impacts tended to report larger effect sizes than those studies that only used self-reported measures [19]. There is also evidence to suggest that for interventions to be most successful they should be targeted at vulnerable individuals who have poor health and live in poor housing. For example, a meta-analysis identified significant health benefits from energy efficiency interventions for vulnerable groups as a whole (e.g. children, the elderly, those on low incomes or pre-existing medical conditions) and for children and people in poor health in particular [19]. The current intervention was targeted at wards with the highest levels of fuel poverty, and the majority of scheme beneficiaries were on low incomes (98% had a household income of less than £16,000) and reported a long-term health condition (90%), which might account for the positive impacts reported by beneficiaries of the programme.

All of the scheme beneficiaries interviewed reported that they were warmer since the work had been completed, which was primarily due to being able to heat their homes to a suitable level of warmth as a result of the installation of new heating systems. As a result people reported using fewer coping strategies to keep warm such as wearing extra layers and using portable heaters. Those people who had children living at home reported that their children also felt warmer, which for them was the most significant impact. In addition, people commented that minor measures such as draught proofing and fixing windows also helped improve the warmth of their home. There was also an indication that those who had both minor and major installations reported a larger increase in both health and wellbeing scores compared to those who just had a major measure, suggesting that a combination of both a minor and major measure has a greater impact on health and wellbeing. This points to the possible importance of comprehensive home energy improvements.

In terms of physical health, some of those interviewed reported fewer respiratory infections and colds, which was attributed to being warmer. Cool temperatures can lower resistance to respiratory infections and therefore increase the risk of respiratory illness [26]. Many people reported that they experienced fewer aches and pain now they were warm as the cold worsened joint pain and arthritic pain. Also, being warmer meant people could move around more, rather than having to sit under blankets, which also helped relieved pain. Furthermore, having hot water meant people could have a hot bath and shower to help relieve joint and muscular pain. Having hot water also meant people were able to wash more regularly, which can impact on physical health (and social activity).

Health impacts of fuel poverty involve more than the direct physical effects of exposure to poor internal conditions. Interview participants reported clear impacts of having new and working heating systems on their mental health and wellbeing. For many these were felt to be the most significant impacts. People described feeling more relaxed, feeling less anxious, and generally happier. These improvements in wellbeing were generally attributed to being less worried about the boiler breaking down or the heating not working. There is also evidence to suggest that the warmth and comfort brought about by the heating installations enhanced a range of psychosocial benefits. For example, an expansion of the domestic space, less worry about bills, improved social interaction and reduced social isolation, feeling less stigma about one’s home, an increase in comfort in the home, and having an increased sense of control over the situation. Although similar findings to these have been reported in several qualitative studies [14, 27], these broader psychosocial and emotional impacts of living in fuel poverty have received less focus compared to physical health impacts. It is important that psychosocial outcomes are considered when examining the impacts of energy efficiency interventions as they are likely intermediary indicators for the potential of long-term health impacts.

Many of the beneficiaries saw a reduction in their energy bills which suggests that the intervention made a difference to feelings of anxiety associated with this aspect of fuel poverty. However, one person in this study reported that despite heating improvements they were still unable to afford to heat their home. As such it is clear that energy efficiency improvements alone are unlikely to address the issue of fuel poverty. Indeed, England’s 2015 fuel poverty strategy has been criticised for framing fuel poverty as an issue solely of energy efficiency and ignoring income inequality and vulnerability more generally [11]. This is in line with an increasing body of research which seeks to widen the conceptualisation of fuel poverty [28]. The majority of this research is guided by the concept of ‘energy vulnerability’, which highlights the multi-dimensional nature of fuel poverty and recognises a range of factors as significant in the production of fuel poverty [11]. For example, Middlemiss and Gillard identify quality of building fabric, tenancy relations, energy cost and supply, stability of household income, social relations, and ill health as important components of fuel poverty [28].

The findings from the current study are broadly consistent with current models of the pathways between fuel poverty interventions and improved physical and mental health [8, 29, 30]. For example, Willand et al. suggest three pathways from energy efficiency interventions to improved health and wellbeing [29]. The “warmth pathway” assumes better energy efficiency will raise indoor temperatures and improve thermal comfort. By reversing the cause of cold related ill-health better warmth is predicted to improve respiratory and cardiovascular health. The “affordability pathway” suggests that energy efficiency interventions will reduce energy consumption and as such fuel costs which could relieve financial stress and subsequently improve mental health. The “psycho-social pathway”, accounts for the psycho-social benefits of energy efficiency interventions, which explains health benefits as a result of enriched meaning of the home.

### Strengths and limitations

A particular strength of the current study is that it used a mixed-methods approach to understand the impacts of the Healthy Homes programme on the health and wellbeing of individuals and families. Previous studies which have explored the impact of energy efficiency interventions have primarily been explored by means of statistical methods, typically focusing on aggregate data. The use of interviews allowed an in-depth exploration of the impacts of the programme on health and wellbeing and the wider social determinants of health, including the variety of circumstance and experiences. Furthermore, the use of quantitative and qualitative methods allows for the triangulation of findings, which can improve the reliability and validity of an evaluation [31]. Finally, the multi-stakeholder evaluation steering group and in particular the inclusion of a steering group member with lived experience of fuel poverty illustrates our inclusive approach to research and ethical practice.

The evaluation nevertheless has some limitations. Firstly, the evaluation lacked a comparator group, which means it is not possible to directly attribute changes identified to the Healthy Homes programme. However, it is recognised that it is very difficult to design a truly comparable group of participants to act as a comparator in such small-scale evaluations [32]. It is suggested that future studies consider a stepped intervention design with a “waiting list control group” [33]. Nonetheless, the current evaluation compared outcomes for beneficiaries of different types of intervention and conducted a qualitative investigation to explore beneficiaries’ experiences of how the programme has impacted on their health and wellbeing; initial literature searches suggest a need for further research on this issue. Secondly, considering the timing of follow-up (approximately 6 weeks since installation) it is possible that there was not sufficient time for significant impact to emerge in the areas of health and wellbeing [34]. Therefore, future evaluations should emphasise the need for monitoring according to protocol and ideally monitor outcomes for longer periods to ensure the full impact of the intervention is observed. Thirdly, it is also important to be aware of the seasonal timing of the baseline and post-installation measures. For example, many respondents reflected on health and wellbeing late spring/early summer and as a result there might be seasonal impacts that cannot be accounted for. These might include impacts on general wellbeing, houses feeling warmer as a result of warmer temperatures outside, and lower energy use. Fourthly, the impact on physical health was only measured using one item (general health) and there was no baseline assessment; only a retrospective measurement of change. Retrospective assessments of health are subject to recall bias due to inaccurate or incomplete recollection, which can result in underestimates or overestimates of earlier health status. Therefore, these findings should be treated with caution. In this study the decision to use the WEMWBS as a tool for collecting generic quality of life data was taken by the providers of the WHCS. Future evaluations could assess health impacts more comprehensively by using a simple subjective health assessment questionnaire (e.g. the EuroQol EQ-5D-5L is recommended in a recent Affordable Warmth and Health Impact Evaluation Toolkit) [35] and/or simple condition-specific questions. Wider health indicators could also be assessed such as days off work and number of visits to health facilities such as GP appointments. Finally, response rates for the questionnaire and interview could be considered low. Only 78 out of the 149 people who received the intervention completed the WEMWBS pre- and post-intervention. The response rate (approximately 52% of all beneficiaries) is similar to a recent evaluation of a fuel poverty intervention which also implemented a postal survey. The current study was conducted in low-income areas, where response rates for postal survey methods tend to be low [36]. Future evaluations should explore alternative recruitment methods to enhance response rates (e.g. contact via phone/SMS reminders) and the uses of incentives to complete the questionnaire. Furthermore, only 23% of scheme beneficiaries participated in an interview, which may suggest selection bias. For example, compared to overall beneficiaries of the scheme participants who took part in the interview were more likely to be home owners. One explanation for this finding might be that home owners were more motivated to provide positive feedback about an intervention that improves a property they own and which might also add value to their property. However, the survey findings did not indicate that there were any differences in health and wellbeing outcomes across tenure. Furthermore, over 80% of the beneficiaries interviewed lived on their own, meaning that it was not possible to explore fully and demonstrate impacts for a household such as the impact on the relationships and dynamics between household members.

## Conclusions

The findings from the study suggest that the installation of major heating or insulation measures such as new boilers and central heating systems have substantial benefits for the health and wellbeing of programme beneficiaries. Although there are limitations to the evaluation design, the consistent message that emerges across all the data adds strength to the evaluation findings. The findings from the qualitative data show that the programme had a positive impact on a number of wider determinants of health including reduction in stress and isolation that are very likely to be part of the pathways between fuel poverty interventions and mental and physical health outcomes. Maintaining social connections, an element of social capital, is identified as a public health priority internationally [37] and in the UK [38] but sometimes the links between improvements in housing and social connections are overlooked or not considered as an outcome. Future research should put more emphasis on the need to explore the broader psychosocial impacts of living in a cold home. For example, the impacts of living in a cold home on the relationship between different household members would be important to explore in further research.

## Data Availability

The qualitative data presented in this manuscript is not publicly available due to the nature of the consent provided by participants for the use of their data. Transcripts of the interviews may be requested from the first author (subject to approval by the university’s ethics committee).

## Abbreviations

ESCC: East Sussex County Council
H&R CCG: NHS Hastings and Rother Clinical Commissioning Group
SAP: Standard Assessment Procedure
WEMWBS: Warwick-Edinburgh Mental Wellbeing Scale
WHCS: Winter Home Check Service

## Footnotes

1
http://www.eastsussexjsna.org.uk/JsnaSiteAspx/media/jsna-media/documents/publichealthreports/2016_17/DPHreport2016_17_Main_report.pdf
